# HER-2 Protein Overexpression in Patients with Gastric and Oesophageal Adenocarcinoma at a Tertiary Care Facility in Ghana

**DOI:** 10.1155/2018/1564150

**Published:** 2018-03-14

**Authors:** David Larbi Simpong, Richard Harry Asmah, Cecilia Krampah, Patrick Kafui Akakpo, Patrick Adu, Du-Bois Asante, Simon Naporo, Andrew Anthony Adjei, Richard Kwasi Gyasi

**Affiliations:** ^1^Department of Medical Laboratory Sciences, University of Cape Coast, Cape Coast, Ghana; ^2^Department of Pathology, University of Ghana, Accra, Ghana; ^3^Department of Medical Laboratory Sciences, University of Ghana, Accra, Ghana; ^4^Department of Pathology, University of Cape Coast, Cape Coast, Ghana; ^5^Institute of Infection, Immunity & Inflammation, University of Glasgow, Glasgow, UK; ^6^Department of Biomedical and Forensic Sciences, University of Cape Coast, Cape Coast, Ghana

## Abstract

The prognosis of gastric and oesophageal adenocarcinoma remains generally poor. However, mounting evidence suggests a positive role of human epidermal growth factor receptor-2 (HER-2) expression in the prognosis of patients with these cancers. In this work, the patterns of HER-2 protein expression were determined in patients with gastric or oesophageal adenocarcinoma. Retrospectively, we reviewed records of gastric and oesophageal biopsies received from 2008 to 2012 and their corresponding archived formalin-fixed paraffin-embedded tissue blocks selected for immunohistochemical analysis. The prevalence of gastric and oesophageal adenocarcinomas and their association with HER-2 protein overexpression were evaluated. Gastric adenocarcinoma made up 18.79% of the gastric biopsies reviewed, and majority of these cancers occurred in males. Regarding the tumour type, HER-2 overexpression was common in the intestinal subtype compared to the diffuse type. Although squamous cell carcinoma was observed to be the commonest (31%) tumour type in the oesophagus compared to adenocarcinoma (8.79%), HER-2 was overexpressed in 42.9% of oesophageal adenocarcinomas, like gastric adenocarcinoma (41.4%). There is a high prevalence of gastric and oesophageal adenocarcinoma, with significant overexpression of HER-2 in these tumours, a window of hope for the management of patients with these cancers.

## 1. Introduction

Gastroesophageal cancer is a major health problem worldwide and is among the leading causes of cancer deaths globally [1]. Although breast, colon, prostate, and lung cancers are more commonly diagnosed cancers, cancers involving the oesophagus and stomach contribute significantly to cancer mortality [1, 2]. Generally, it appears that a small fraction of gastric and oesophageal cancer patients respond to the current management modalities such as surgery, chemotherapy, or radiotherapy. This suggests the need to explore other possible and effective approaches to managing these cancer patients [3, 4]. Increasing evidence suggests an improved prognosis in these cancers when therapy is targeted towards the biomarker, human epidermal growth factor receptor-2 (HER-2) [5]. HER-2, a transmembrane tyrosine kinase, has been shown to have therapeutic and thus prognostic implications in gastric [6, 7] and oesophageal [8] cancers. A significant survival advantage has been identified in patients who overexpress HER-2. It has also been shown that gastric and oesophageal adenocarcinoma patients who overexpress HER-2 benefit from trastuzumab (a HER-2 specific monoclonal antibody), when combined with the traditional treatment regimen [9–11]. It is imperative that patients with these tumours that overexpress HER-2 are selected to benefit from HER-2-targeted therapy.

Presently, routine testing for HER-2 protein overexpression in gastric or oesophageal adenocarcinoma does not occur in Ghana. This means that patients with these tumours that overexpress HER-2 protein are not identified and thus do not benefit from HER-2-targeted therapy. In this study, we explored the local pattern of adenocarcinoma of the stomach and oesophagus and its association with HER-2 overexpression within a period of five years using archived 10% buffered, formalin-fixed, paraffin-embedded tissue blocks.

## 2. Materials and Methods

### 2.1. Data Collection

This was a retrospective study involving archived 10% buffered, formalin-fixed, paraffin-embedded tissues with well-documented records in the books of Pathology Department, Korle Bu Teaching Hospital (KBTH), Ghana, from 2008 to 2012. KBTH is tertiary care hospital in Ghana where majority of cases within the country and some cases from neighbouring west African countries are referred. All specimens were from individuals who suffered from either oesophageal or gastric cancer. Histopathologically, 183 cases were diagnosed as adenocarcinoma of the stomach, and 8 were diagnosed as oesophageal adenocarcinoma within the selected period. 99 out of the 183 gastric cancers and all of the 8 oesophageal cancers had sufficient tumour burden to allow further objective analysis. Both excision specimens and endoscopic biopsy specimens were used. The study was approved by the Ethical and Protocol Review Committee, University of Ghana Medical School, College of Health Science, Korle Bu, Accra, Ghana.

### 2.2. Tissue Processing

Paraffin-embedded tissue blocks were individually sectioned to a thickness of 4 *μ*m. Sections representing a full phase of the tissue blocks were stained with Haematoxylin and Eosin (H&E). The Lauren classification system [12] was used to classify the gastric adenocarcinoma into diffuse and intestinal subtypes. Tumours with single cells or poorly differentiated cells that diffusely infiltrate the wall of the stomach as well as signet ring cell carcinoma were all classified as diffuse type, while the remaining adenocarcinomas were classified as intestinal type. Other tumour types, such as non-Hodgkin's lymphoma, gastrointestinal stromal tumour (GIST), and carcinoid tumour, were all excluded from the study. Immunohistochemistry (Novolink™ staining technique, Leica Microsystems, UK) was used to detect the expression of HER-2 after antigen retrieval with the aid of a pressure cooker.

### 2.3. The Scoring of HER-2 Expression

Evaluation and scoring of HER-2 protein overexpression were performed according to the criteria proposed by Rüschoff et al. [13]. HER-2 antibody was purchased from Leica Microsystems (UK). HER-2 expression was scored as follows: 0 (negative), no reactivity or no membranous reactivity in any tumour cell; 1+ (negative), tumour cell cluster with faint/barely perceptible membranous reactivity irrespective of the percentage of tumour cells stained; 2+ (equivocal), tumour cell cluster with a weak-to-moderate, complete, basolateral, or lateral membranous reactivity irrespective of the percentage of tumour cells stained; 3+ (positive), tumour cell cluster with a strong complete, basolateral, or lateral membranous reactivity irrespective of the percentage of tumour cells stained. A tumour was HER-2-positive when a score of 3+ was assigned and negative when a score of 0 or 1+ was graded. Equivocal score (2+) for HER-2 was excluded from this study. Slides were first screened and later reviewed independently by a consultant pathologist. Discordant cases were reviewed together and a final consensus was reached.

### 2.4. Data Analysis

Data was entered into an Excel spreadsheet (Microsoft Company) and analysed using SPSS version 16 and Minitab. Continuous variables were tested for homogeneity of variances and normality before analysis. Appropriate measures of centrality (mean, median) and of dispersion (standard deviation) were calculated. Graphical displays such as frequency distributions were created where appropriate. Frequencies and percentages were calculated for dichotomous data such as HER-2. For hypotheses comparing frequencies among groups (age, gender, and tumour type), Fisher's exact test was used to test their association with the biomarker, HER-2. All statistical tests were two-tailed and a *p* value less than 0.05 was interpreted as significant.

## 3. Results

**Figure 1:**
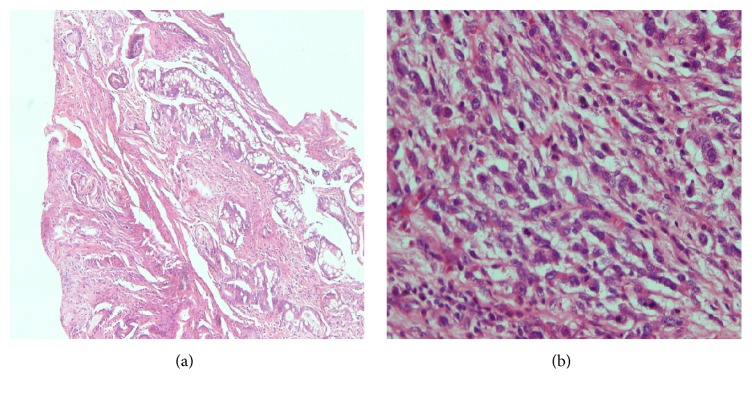
Haematoxylin and eosin stained sections showing adenocarcinoma. (a) Intestinal type gastric adenocarcinoma showing invasion of the muscularis propria by moderately differentiated malignant glands. (b) Diffuse type gastric adenocarcinoma showing monomorphic tumour cells with an Indian-file pattern of infiltration (magnification ×200).

Over the 5-year period, 974 primary gastric biopsies with patients' biographical records such as age and gender were retrieved from the histopathology log books. 183 (18.79%) of these gastric biopsies were diagnosed as adenocarcinoma, 99 of which had sufficient material to allow objective further analysis. 22.2% out of the 99 were subclassified as diffuse type while the remaining 77.8% (77 samples) were subclassified as intestinal type. Figures 1(a) and 1(b) are, respectively, representative photomicrographs of intestinal and diffuse histomorphological types of adenocarcinoma.

The demographic characteristics (age, gender, and histologic subtypes) of the 99 gastric adenocarcinoma subjects are shown in Table 1. 41 of these subjects overexpressed the biomarker, HER-2. The 99 gastric adenocarcinoma subjects were aged 22–100 years (mean age: 59.5 ± 13.91 SD). The lowest and highest rates of gastric adenocarcinoma were noticed in the age groups of <55 years and 55–100 years, respectively. Of the study subjects, 59.6% (59 out of 99) were females, aged 22 to 100 years (mean: 57.9 ± 15.53 SD), whereas the remaining were males, aged 25 to 80 years (mean: 60.04 ± 12.85).

There was an interesting association between HER-2 overexpression and age or gender of the participants (Table 1). 28 out of 41 subjects who overexpressed HER-2 were ≥55 years of age, whereas 13 were below 55 years of age. A significant association (*p* = 0.034) was observed between participant age groups and HER-2 overexpression (Table 1).

**Figure 2:**
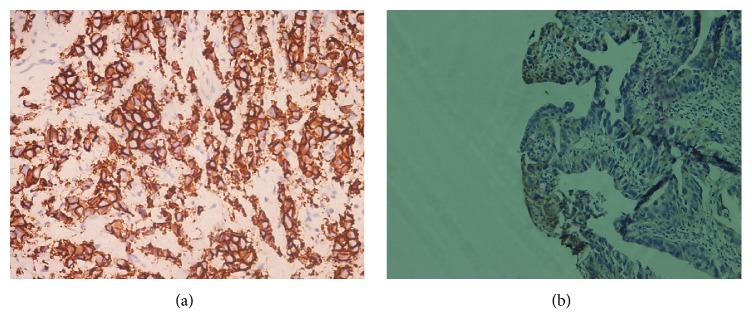
Photomicrographs of immunohistochemical staining: (a) HER-2-positive (magnification ×400) and (b) HER-2-negative (magnification ×200).

20 participants out of the 41 subjects who overexpressed HER-2 were males and the remaining 21 were females. There was no significant association between gender and HER-2 expression (*p* > 0.05, Table 1). 34 out of 41 subjects who overexpressed HER-2 had intestinal type gastric adenocarcinoma, and 7 out of the remaining 41 cases were of a diffuse type. HER-2 overexpression was significant (*p* = 0.001) among the tumour subtype of gastric adenocarcinomas. Figures 2(a) and 2(b) show photomicrographs of HER-2 positivity (3+) and negativity, respectively.

Among the 91 evaluable oesophageal biopsies (36–94 years; median age: 45 years), 8 cases representing 8.79% were adenocarcinoma. Of these 8 cases, 3 were females (median age: 43 years) and 5 males (median age: 48 years), and only 3 overexpressed HER-2 (2 males and 1 female) and were aged ≥40.

## 4. Discussion

Gastric and oesophageal adenocarcinomas are leading causes of cancer death worldwide. Presently, a lot of effort is being made to understand specific biomarkers that could be of prognostic value to improve the survival rate of patients. We investigated the pattern of gastric and oesophageal adenocarcinomas as well as their HER-2 expression in patients that visited a tertiary care hospital in Ghana over a period of 5 years.

Of the total 974 gastric excisions and endoscopic biopsies received during the 5-year period, approximately 18.79% (183 out of 974) were diagnosed with gastric adenocarcinoma. Although 99 cases out of these 183 gastric adenocarcinoma cases had sections representing a full phase of the tissue blocks and therefore were selected for immunohistochemical analysis, the prevalence recorded in this study requires prompt care. Regarding geographical locations, there appears to be a variation in the prevalence of this tumour, suggesting the relevance of the study within a locality. For instance, in Portugal, 10.3% of patients were found to have gastric adenocarcinoma out of a total of 1434 patients [14]. This was a typical illustration of variations in the prevalence of this tumour at a given place, hence the need for this study to present the pattern of the disease in Ghana and inform prompt attention. Based on our study, it is tempting to suggest that gastric adenocarcinomas are of high prevalence and need swift consideration. Several factors have been cited as potential risk factors for the prevalence of gastric adenocarcinoma. These risk factors may include* Helicobacter pylori*, human papilloma virus, and Epstein-Barr virus infections [15–17]. In addition, the method of food preservation has also been shown to play a crucial role in the variation of gastric tumour pattern [18]. Although this study did not address the risk factors underlying the observed cases of gastric adenocarcinoma in the Ghanaian setting, it will be interesting to investigate which risk factors strongly associate with gastric adenocarcinoma to better inform the populace and potentially reduce the incidence of this cancer through lifestyle changes.

Globally, gastric cancer is a disease of the aged population with preponderance in men [19]. In agreement with other previous reports that found a mean age of 60 ± 15 SD years [1, 20], we also found an increasing age (59.95 ± 15 SD) and male predominance (61.7%) in this study. Accumulation of genetic defects and excessive intake of salty food [21] have been suggested as a possible explanation for the disease being more frequent in the elderly and common in men. The significant association of gastric adenocarcinoma and the male gender raises the question of whether hormonal influences have a role in the aetiology of the disease. Moreover, the distribution of diffuse type gastric adenocarcinoma appears to have a similar pattern globally. The intestinal variant appears to be more affected by geographical location and is the more common histomorphologic variant of gastric adenocarcinoma [22, 23]. We also found a similar pattern in this study.

The pattern of HER-2 overexpression in gastric adenocarcinoma has been noted in several studies to range from 9% to 38% [6, 22, 24–26]. However, in this study, 41.4% (41 of 99 patients) gastric adenocarcinoma cases were HER-2-positive (3+) by immunohistochemistry. A previous study showed that, in gastric adenocarcinoma, HER-2 positivity differed significantly by histological subtype, 34% being intestinal and 6% being diffuse [23]. Here, we observed a similar pattern with predominance of the intestinal subtype (77.8%) compared to the diffuse subtype (22.2%). The reasons for these diversities may be genetically or environmentally linked and therefore warrant further study in the Ghanaian situation.

This study also found some interesting data on oesophageal adenocarcinoma in the Ghanaian setting. The prevalence of oesophageal adenocarcinoma among 91 oesophageal biopsies examined was 8.79%. Previous studies have shown variations in the incidence of oesophageal cancers in both sexes. For example, an important variation in the male-to-female ratio has been recorded in different settings to range from 0.85 in Northern Iran [27] to 20.5 in Hispanics [28] for this cancer. Similarly, we also found oesophageal adenocarcinoma to be higher in males compared to females in this study. A possible suggestion for the variation in this tumour is likely to be a genetic link [29]. The observed higher prevalence in males may also be associated with excessive smoking of tobacco [30] by men compared to women. However, the joint effect of alcohol and smoking when consumed collectively may potentiate oesophageal adenocarcinoma in males, an interesting area that can be explored.

In sub-Saharan Africa, oesophageal adenocarcinoma was prominent among people of both sexes aged 45–65 years [31]. This is comparable in both developed and developing countries. Adenocarcinoma of the oesophagus is infrequent before the age of 40, beyond which the frequency increases [32]. Regarding subjects with oesophageal adenocarcinoma in this study, a mean age of 47.7 years was observed. HER-2 protein expression in oesophageal adenocarcinoma has been noted globally [23]. Interestingly, out of the 9 oesophageal adenocarcinoma subjects, 3 overexpressed HER-2. Although this cancer appears not to be common, it is important to explore possible management options to contain it ahead of time. Besides the fact that these findings in our study provide the baseline for further research, it also suggests the importance of screening for HER-2 expression in patients with either gastric or oesophageal adenocarcinoma.

## 5. Conclusion

In conclusion, our data shows a high prevalence of gastric and oesophageal adenocarcinoma, with significant overexpression of HER-2 in these tumours in the Ghanaians. Ghanaian patients with oesophageal or gastric adenocarcinoma should be screened for the expression of HER-2.

## Figures and Tables

**Table 1:** Human epidermal growth factor receptor-2 (HER-2) overexpression in gastric adenocarcinoma.

Parameters	Patient (*n*)	HER-2-positive patients (*n*)	*p* value (*α* = 0.05)

*Age*			
0–14	0	0	
15–44	13	6	
45–54	18	7	0.034
55–64	29	10	
>64	39	18	
*Gender*			
Male	40	20	0.655
Female	59	21	
*Tumour type*			
Intestinal	77	34	0.001
Diffuse	22	7	
